# Sporadic medullary thyroid carcinoma with a rare *RET* transmembrane domain mutation (A641R) that responds to selpercatinib

**DOI:** 10.1093/oncolo/oyaf209

**Published:** 2025-07-10

**Authors:** Naoki Fukuda, Kazuhisa Toda, Tomohiro Chiba, Ippei Fukada, Seiichi Mori, Akiko Tonooka, Erisa Toda, Jumpei Yoshida, Xiaofei Wang, Ryosuke Oki, Takehiro Nakao, Tetsuya Urasaki, Kenji Nakano, Makiko Ono, Junichi Tomomatsu, Kengo Takeuchi, Shunji Takahashi, Yuji Miura

**Affiliations:** Department of Medical Oncology, The Cancer Institute Hospital of Japanese Foundation for Cancer Research, Tokyo 135-8550, Japan; Department of Clinical Cancer Genomics, Hokkaido University Graduate School of Medicine, Sapporo 060-8638, Japan; Department of Head and Neck Oncology, The Cancer Institute Hospital of Japanese Foundation for Cancer Research, Tokyo 135-8550, Japan; Department of Pathology, The Cancer Institute Hospital of Japanese Foundation for Cancer Research, Tokyo 135-8550, Japan; Department of Genomic Medicine, The Cancer Institute Hospital of Japanese Foundation for Cancer Research, Tokyo 135-8550, Japan; Department of Genomic Medicine, The Cancer Institute Hospital of Japanese Foundation for Cancer Research, Tokyo 135-8550, Japan; Division of Cancer Genomics, Cancer Precision Medicine Center, Japanese Foundation for Cancer Research, Tokyo 135-8550, Japan; Department of Genetic Diagnosis, Cancer Institute Hospital, Japanese Foundation for Cancer Research, Tokyo 135-8550, Japan; Department of Pathology, The Cancer Institute Hospital of Japanese Foundation for Cancer Research, Tokyo 135-8550, Japan; Department of Medical Oncology, The Cancer Institute Hospital of Japanese Foundation for Cancer Research, Tokyo 135-8550, Japan; Department of Medical Oncology, The Cancer Institute Hospital of Japanese Foundation for Cancer Research, Tokyo 135-8550, Japan; Department of Medical Oncology, The Cancer Institute Hospital of Japanese Foundation for Cancer Research, Tokyo 135-8550, Japan; Department of Medical Oncology, The Cancer Institute Hospital of Japanese Foundation for Cancer Research, Tokyo 135-8550, Japan; Department of Medical Oncology, The Cancer Institute Hospital of Japanese Foundation for Cancer Research, Tokyo 135-8550, Japan; Department of Medical Oncology, The Cancer Institute Hospital of Japanese Foundation for Cancer Research, Tokyo 135-8550, Japan; Department of Medical Oncology, The Cancer Institute Hospital of Japanese Foundation for Cancer Research, Tokyo 135-8550, Japan; Department of Medical Oncology, The Cancer Institute Hospital of Japanese Foundation for Cancer Research, Tokyo 135-8550, Japan; Department of Medical Oncology, The Cancer Institute Hospital of Japanese Foundation for Cancer Research, Tokyo 135-8550, Japan; Department of Pathology, The Cancer Institute Hospital of Japanese Foundation for Cancer Research, Tokyo 135-8550, Japan; Department of Medical Oncology, The Cancer Institute Hospital of Japanese Foundation for Cancer Research, Tokyo 135-8550, Japan; Department of Genomic Medicine, The Cancer Institute Hospital of Japanese Foundation for Cancer Research, Tokyo 135-8550, Japan; Department of Medical Oncology, The Cancer Institute Hospital of Japanese Foundation for Cancer Research, Tokyo 135-8550, Japan

**Keywords:** medullary thyroid cancer, RET, selpercatinib, proto-oncogene, protein-tyrosine kinase

## Abstract

Medullary thyroid carcinoma is a rare thyroid malignancy derived from parafollicular C cells that is frequently driven by activating mutations in the REarranged during Transfection (*RET*) proto-oncogene. While most actionable *RET* mutations are located in the extracellular cysteine-rich or intracellular tyrosine kinase domains, mutations in the transmembrane domain are exceedingly rare and their oncogenic significance remains unclear. We report a case of a 59-year-old male with sporadic medullary thyroid carcinoma harboring a rare *RET* A641R mutation in the transmembrane domain. The patient experienced multiple locoregional recurrences after four surgical resections. While the companion diagnostic test did not identify *RET* mutations, comprehensive genomic profiling using a next-generation sequencing panel revealed the *RET* A641R mutation. Following administration of selpercatinib, a selective RET inhibitor, a rapid biochemical response with decreased serum carcinoembryonic antigen and calcitonin levels was observed, and radiological assessment showed partial response. This is the first report demonstrating the clinical efficacy of selpercatinib in a patient with medullary thyroid carcinoma harboring a *RET* A641R mutation, supporting the oncogenic potential of this rare variant. This case also emphasizes the importance of comprehensive genomic profiling in identifying rare but actionable *RET* alterations that are undetectable by targeted sequencing companion diagnostic tests. Selpercatinib may represent an effective therapeutic option for patients with medullary thyroid carcinoma driven by uncommon *RET* mutations, including mutations in the transmembrane domain.

## Graphical abstract

**Graphical Abstract:**
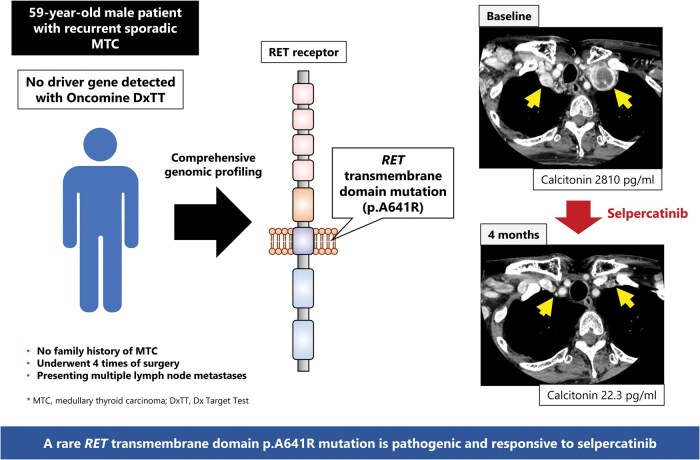

Glossary of genomic terms and nomenclature

RET, REarranged during Transfection

Key Points

- In many cases of medullary thyroid carcinoma (MTC), mutations in the RET gene serve as driver mutations, with the majority found in the cadherin-like domain, the cysteine-rich domain, and the intracellular tyrosine kinase domain.
- This report is the first to provide evidence that the rare A641R mutation in the transmembrane domain of RET functions as an oncogenic driver in MTC. This report also indicates that selpercatinib, a selective RET inhibitor, is effective in patients with MTC harboring this mutation.
- Because mutations in the transmembrane domain of RET are rare and typically not included in the companion diagnostic panels using targeted sequencing, comprehensive genomic profiling is recommended when common genetic alterations are not detected in patients with MTC.

## Introduction

Medullary thyroid cancer (MTC) is a rare form of thyroid carcinoma that originates in the parafollicular C cells of the thyroid gland.^1^^,^^2^ MTC occurs both sporadically and as part of hereditary cancer syndromes, including multiple endocrine neoplasia (MEN) types 2A and 2B and familial MTC^3^^,^^4^.

The *REarranged during Transfection (RET)* gene, a member of the cadherin superfamily, encodes a membrane receptor tyrosine kinase. RET was the first activated receptor tyrosine kinase identified in thyroid neoplasms. Activating mutations in the *RET* gene are implicated in both sporadic and hereditary MTC, including MEN2A, MEN2B, and familial MTC.^5–7^ In MEN2A, which accounts for 90%-95% of MEN2 cases, *RET* mutations are detected in over 90% of patients.^8^ The *RET* gene consists of 21 exons located on chromosome 10 (10q11.2). The RET receptor protein consists of a cadherin-like domain, a cysteine-rich domain, a transmembrane domain, and an intracellular tyrosine kinase domain. Exons 10 and 11 encode the extracellular cysteine-rich domain, whereas exons 13, 14, 15, and 16 encode the intracellular tyrosine kinase domain.^9^ The common *RET* mutations in MEN2A are located in exon 10 (codons 609, 611, 618, or 620), exon 11 (codons 630 or 634), exon 13 (codons 768 or 790), exon 14 (codon 804), or exon 15 (codon 891). In contrast, the majority (>95%) of MEN2B cases harbor the M918T mutation in exon 16.^10^ In sporadic MTC, most cases are associated with the *RET* M918T (exon 16) or C634 (exon 11) mutations.^11^

For recurrent or metastatic MTC, systemic chemotherapy is a therapeutic option, particularly for patients with progressive disease. In the phase 1/2 LIBRETTO-001 trial, the selective RET inhibitor selpercatinib demonstrated a favorable response rate and improved progression-free survival (PFS) in *RET* mutation-positive MTC or fusion-positive thyroid cancers.^12^ Furthermore, selpercatinib resulted in prolonged PFS compared with conventional standard multi-kinase inhibitors in patients with treatment-naïve *RET* mutation-positive MTC in the phase 3 randomized controlled trial (LIBRETTO-531). Although adverse events such as hypertension, diarrhea, elevated transaminase levels, QT interval prolongation on electrocardiogram (ECG), edema, dry mouth, and erectile dysfunction were observed with selpercatinib, the incidence of grade ≥3 adverse events was lower than with multi-kinase inhibitors.^13^ Consequently, selpercatinib is now regarded as the standard systemic therapy for *RET* mutation-positive MTC.

Unlike *RET* mutations in the cysteine-rich and intracellular tyrosine kinase domains, *RET* mutations in the transmembrane domain are rarely reported, and the oncogenic significance of these mutations remains poorly understood. Moreover, there are very few reported cases describing the efficacy of selpercatinib for patients with MTC harboring *RET* mutations in the transmembrane domain. Here, we present the first case of sporadic MTC with the *RET* A641R transmembrane domain mutation, which responded to selpercatinib treatment.

## Patient story

A 59-year-old man was referred to our hospital for a detailed examination of a thyroid tumor. Computed tomography (CT) imaging revealed a tumor in the right thyroid lobe and multiple right cervical lymph node metastases. No distant metastases were identified. Fine needle aspiration cytology of the thyroid tumor suggested MTC. Genetic screening of a blood sample confirmed the absence of germline *RET* mutations. The patient’s past medical history was unremarkable. The patient did not have a family history of MTC or other neuroendocrine neoplasms.

**Figure 1.**
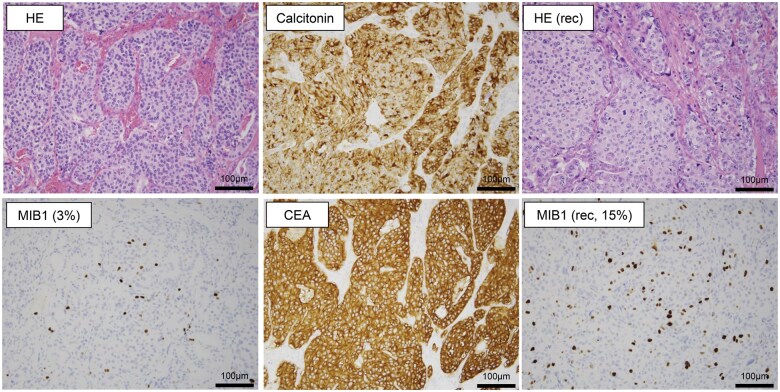
Histopathologic examination of the primary site and recurrent site (rec). hematoxylin & eosin (HE) staining and immunohistochemistry with antibodies against calcitonin, CEA, and Ki-67 (MIB1) were performed. Histological and immunohistochemical staining of the thyroid tumor and recurrent site of the patient in this case report.

The patient underwent a right thyroid lobectomy and D2b lymph node dissection, which confirmed a diagnosis of pT3N1bM0 MTC (Figure 1). Two years later, he underwent a second surgery including total thyroidectomy and lymph node dissection to address right cervical lymph node and left superior mediastinal lymph node metastases. One year later, thoracoscopic mediastinal lymph node dissection was performed for tumor debulking of mediastinal lymph node metastases. A fourth surgery involving right cervical node dissection was performed one year later. Histopathologically, the case tended to exhibit increased cellular atypia and a higher MIB1 index with repeated recurrences (Figure 1).

In 2022, selpercatinib was approved for *RET* mutation-positive MTC in Japan. Consequently, an Oncomine Dx Target Test (DxTT) (Thermo Fisher Scientific, Waltham, MA, USA) was performed using a specimen from the fourth surgery as a companion diagnostic test for selpercatinib. The results revealed no *RET* mutations. Additionally, no other mutations, including *RAS* or *PIK3CA* mutations, were detected. The patient then underwent comprehensive genomic profiling using a next-generation sequencing (NGS) panel, GenMine TOP (Konica Minolta REALM, Inc., Tokyo, Japan), to identify potential druggable genetic alterations. This panel assesses mutations in 737 cancer-related genes and 455 cancer-related fusion genes, exon skipping in 5 cancer-related genes, gene expression levels of 27 cancer-related genes, and tumor mutational burden. The results identified a *RET* A641R mutation (variant allele frequency [VAF], 28.3%), an *H3-3B* R9C mutation (VAF, 39.3%), and an *XPO5* R519Q mutation (VAF, 25.6%) (estimated tumor cell content, 70.0%) (Table 1). The tumor mutational burden was low, measured at 1.6 mutations per megabase pair.

**Table 1.** Results from next-generation sequencing panel (GenMineTOP, Konica Minolta REALM, Inc., Tokyo, Japan).

Gene	Reference sequence	c.HGVS; p. HGVS	Allele frequency, %
***RET***	NM_020975.6	c.1921_1922delinsCG; p. A641R	28.3
***H3-3B***	NM_005324.5	c.25C>T; p. R9C	39.3
***XPO5***	NM_020750.3	c.1556G>A; p. R519Q	25.6

Abbreviation: HGVS, Human Genome Variation Society.

## Molecular tumor board

The *RET* M918T mutation is the most commonly detected *RET* mutation in advanced MTC cases.^14^^,^^15^ Most *RET* mutations, including M918T, are found in the extracellular cysteine-rich domain or the intracellular tyrosine kinase domain. In contrast, actionable mutations in the transmembrane domain are rarely reported.^9^

Ligand binding leads to the dimerization of the RET protein, which results in autophosphorylation of tyrosine residues in the tyrosine kinase domain and subsequently activates intracellular signaling cascades. In patients with *RET* mutations in the extracellular cysteine-rich domain, dimerization of the RET protein occurs independently of ligand binding, resulting in constitutive signal activation.^16^ Previous studies have suggested a potential role of the RET transmembrane domain in RET protein dimerization.^17^ Three amino acid residues (A641, S649, and S653) are responsible for the self-association of the transmembrane domain, which is essential for receptor dimerization.^18^ Among these mutations, mutations at *RET* A641 are rarely reported in MTC. A germline double mutation of A641S/C634S in a patient with MEN2A was identified in 2005 and a single germline A641T mutation was reported in 2015.^19^^,^^20^ Regarding the A641R variant in the present case, a double mutation of A639G/A641R in a patient with sporadic MTC was first reported in 2001.^21^ Another study indicated that both the A641R single mutation and the A639G/A641R double mutation reduced the self-association of the RET transmembrane domain. The authors concluded that the A639G/A641R mutation does not support MTC tumor formation.^18^ Therefore, the oncogenic impact of a single *RET* A641R mutation in sporadic MTC cases had not been demonstrated.

Although the *RET* A641R variant was not listed in major databases such as ClinVar or COSMIC, we decided to administer selpercatinib to this patient. This decision was based on evidence from a phase III trial, which demonstrated significantly longer PFS with selpercatinib as a first-line treatment compared to multi-kinase inhibitors such as vandetanib or cabozantinib.^13^ Given the possibility that this genetic alteration may be pathogenic, we were concerned that the patient might miss the opportunity to receive a potentially effective and less-toxic therapy. Furthermore, because the lymph node metastasis was located adjacent to major cervical vessels, the use of multi-kinase inhibitors with vascular endothelial growth factor-inhibitory activity was considered less feasible due to the associated risk of bleeding.

## Patient update

**Figure 2.**
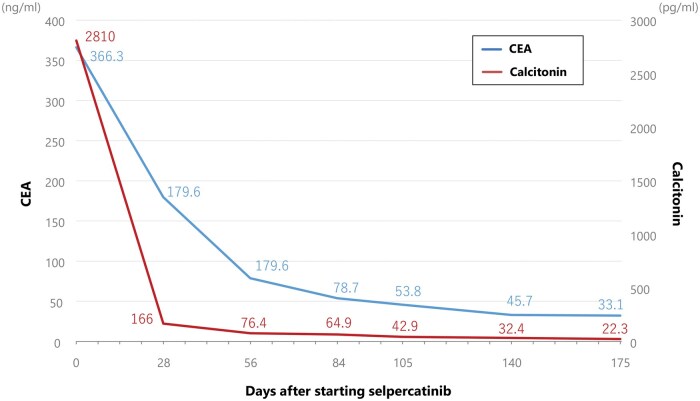
Plasma levels of CEA and calcitonin before and after commencement of selpercatinib. Graph depicting the trends of CEA and calcitonin levels in the patient treated with selpercatinib.

**Figure 3.**
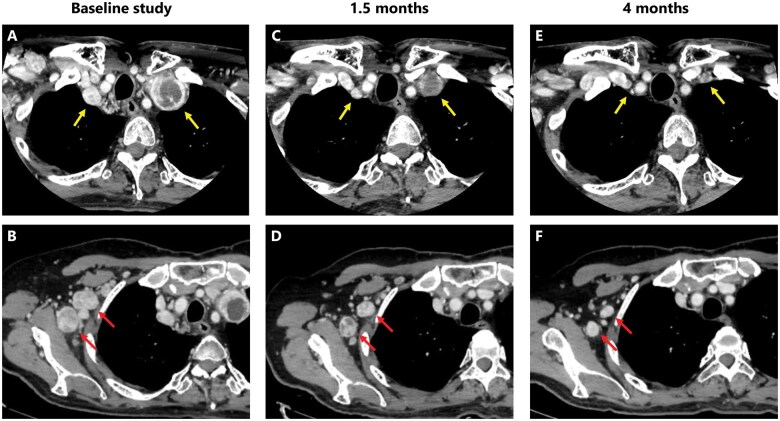
Representative axial, enhanced computed tomography pictures of cervical and axillary lymph node metastases (arrows) taken just before (A, B), 1.5 months after (C, D), and 4 months after (E, F) commencement of selpercatinib. Computed tomography imaging findings of metastases in the patient treated with selpercatinib, with subpanels labelled from A to F.

On the basis of these considerations, the patient received treatment with selpercatinib (160 mg orally, twice daily) for progressive multiple cervical and axillary lymph node metastases. Serum CEA and calcitonin levels rapidly decreased following the initiation of selpercatinib (Figure 2). Contrast-enhanced CT imaging revealed marked tumor reduction, meeting the criteria for partial response under Response Evaluation Criteria in Solid Tumors (Figure 3). Aside from a Grade 1 QT interval prolongation noted on ECG, no other clinically relevant adverse events were observed.

This is the first documented case of a patient with MTC harboring a *RET* transmembrane domain A641R mutation that showed response to the selective RET inhibitor selpercatinib. Marked tumor reduction was achieved with selpercatinib, suggesting the oncogenic relevance of the *RET* A641R mutation in MTC. This case also highlights the anti-tumor effect of selpercatinib in patients with *RET* transmembrane domain mutations. Most *RET* mutations in MTC occur in the extracellular cysteine-rich domain or the intracellular tyrosine kinase domain, and *RET* minor mutations were not described in detail in the pivotal published studies.^12^^,^^13^ The efficacy of selpercatinib for uncommon *RET* mutations was recently reported through a combined data analysis of two critical studies. Clinical response to selpercatinib in patients with *RET* transmembrane domain mutations (codons 636–657) was reported in only three cases (C636_V637insCRT, S649L, and S649_V650insLLLL) out of 509 analyzed cases (0.6%).^22^ The A641R mutation was not detected in that study, making the present case the first report of a patient with MTC harboring *RET* A641R mutation who responded to selpercatinib.

In this case, the *RET* A641R mutation was not detected by the companion diagnostic test (Oncomine DxTT) but was successfully identified by the NGS panel (GenMine TOP). Oncomine DxTT is a targeted NGS panel and not designed to detect RET minor mutations, including A641R. In sporadic MTC, 45%-70% of cases involve *RET* activating mutations and 10%-45% of cases involve *RAS* mutations.^23^^,^^24^ MTC with both *RET* and *RAS* wild-type is relatively rare, with a reported incidence of less than 10%.^25^ Therefore, when both *RET* and *RAS* mutations are absent in patients with MTC evaluated by companion diagnostic tests using targeted NGS, physicians should consider further comprehensive genomic profiling to identify minor *RET* alterations and provide potential opportunities for effective treatment.

## Conclusion

Although exceptionally rare, the *RET* transmembrane domain A641R mutation can act as an oncogenic driver in MTC. The RET-selective inhibitor selpercatinib is a valid treatment option for patients with MTC harboring this rare mutation and those with other major mutations. Physicians should be aware of the importance of using comprehensive genomic profiling to identify rare mutations and improve outcomes for MTC patients.

## Data Availability

The data cannot be shared publicly because of ethical restrictions. The data will be shared on reasonable request to the corresponding author.
